# Tuning TiO_2_ Memristors by Defect Engineering: From Short-Term Memory to Recoverable Long-Term Resistance States

**DOI:** 10.1021/acsami.6c04844

**Published:** 2026-05-05

**Authors:** Rajdeep Kaur, Tuan Thien Tran, Rebecka Lindblad, Zhen Zhang, Daniel Primetzhofer, Petter Ström

**Affiliations:** † Division of Materials Physics, Department of Physics and Astronomy, 8097 Uppsala University, 751 20 Uppsala, Sweden; ‡ Division of X-ray Photon Science, Programme of Condensed Matter Physics of Energy Materials, Department of Physics and Astronomy, Uppsala University, 751 20 Uppsala, Sweden; § Division of Solid-State Electronics, Department of Electrical Engineering, Uppsala University, 751 20 Uppsala, Sweden; ∥ Tandem Laboratory, Uppsala University, 751 21 Uppsala, Sweden

**Keywords:** ion implantation, TiO_2_, oxygen vacancy, conductive filament, electroforming-free resistive switching

## Abstract

Resistive switching memory cells (memristors), with potential applications in neuromorphic computing and next-generation data storage, were studied. Sputter-deposited samples of Pd/TiO_2_/Pd memristor structures exhibited bipolar switching with long-term (nonvolatile) memory after an initial electroforming process. The samples were subsequently implanted with 8 keV ^16^O and ^20^Ne ions to tune switching properties by controlled introduction of defects in the TiO_2_ layer. A modification in the oxygen vacancy concentration resulting from the implantation was verified using X-ray photoelectron spectroscopy with both Al and Ga Kα X-rays. Ion implantation enabled electroforming-free RS with short-term (volatile) memory and weighted resistance states with long-term memory, which can be modulated via compliance current, in addition to the RS with long-term memory exhibited by the as-prepared samples. The potential underlying mechanism for switching in ion-implanted memristors is discussed, relating it to the tuning of switching properties through ion implantation. Ion-implanted samples, which exhibited electroforming-free short-term memory, showed a decrease in failure rate. These findings highlight the potential of ion implantation as a versatile tool for tuning switching characteristics of memristors.

## Graphical abstract

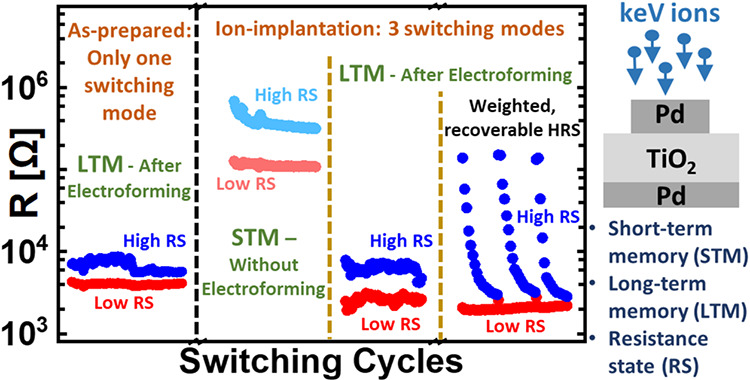

## 1 Introduction

With rapidly evolving information technology and artificial intelligence (AI) on one hand, and complementary metal-oxide semiconductor (CMOS) scaling approaching its physical constraints on the other, the search for more innovative solutions for data storage and computation has become critical.^1,2^ One of the emerging solutions is resistive switching-based memory cells, consisting of a simple metal–insulator–metal (MIM) configuration.^3,4^ These devices exhibit promising properties for next-generation memory devices: the ability to store and compute simultaneously, low cost, high packing density, low power consumption, fast switching speed, scalability and compatibility with CMOS technologies.^5^ The resistive switching (RS), first reported by Hickmott in 1962,^6^ is a reversible change in the resistance of a system under external stimuli, such as voltage or current. An RS memory cell can be described as a memristor, which was postulated as the fourth fundamental circuit element by Chua in 1971.^7^ The first functional nanoscale memristor device (Pt/TiO_2_/Pt) was developed by HP in 2008.^8^ In addition to data storage, RS-based devices are also being studied for potential applications in other fields, such as neuromorphic computing,^9,10^ programmable circuits,^11^ and hardware security.^9^

Although resistive switching devices show great potential, they face significant challenges, such as variability and degradation over time and switching cycles, to be commercially viable.^5,10,11^ Different insulating materials, such as transition metal oxides (e.g., TiO_*x*_, HfO_*x*_ and NbO_*x*_),^12^ complex oxides (e.g., perovskites),^13^ chalcogenides (e.g., Ag_2_S, GeSe and In_2_Te_3_),^14^ and polymers (e.g., polyaniline),^15^ are being investigated. Recently, multilayered insulating layers^16,17^ and two-dimensional materials^13,18^ have also been widely studied. It has been reported that the switching mechanism and performance depend on both the insulating layer and the electrodes.^12,19–22^ The electrodes can be broadly divided into three categories based on the degree to which electrode material is physically or chemically involved in the switching: inert (e.g., Pt and Pd), relatively active (e.g., Ti and W) and active (e.g., Ag and Cu).^19,23^ The roughness of the interface layers can also play a critical role in the resistive switching properties. A significant decrease in the surface roughness of the bottom electrode could lead to higher electroforming voltages, an increase in device failure rates and the formation of weaker conductive filaments, thereby hampering the performance of the memristors.^22,24,25^ Compliance current (*I*_cc_) is also reported to play an important role in switching properties such as resistance states and memory retention.^26–29^ Several mechanisms have been proposed for resistance switching in MIM structures: filament formation and rupture due to migration of metal ions or oxygen vacancies in the insulating layer, phase transition in the insulating layer, Joule heating, charge trapping/detrapping, and tunneling.^20,30,31^

TiO_2_ is a low-cost, high-k dielectric material used in plastics, paints, catalysts, sunscreens, inks, and water purification and disinfection. It is also widely studied for resistive switching, exhibiting both unipolar and bipolar switching.^12,16,32,33^ The resistance of the initially insulating MIM structure can be reduced by applying voltage or current. This initialization process for switching resistance is called electroforming. In the case of transition metal oxide memristors with inert electrodes (similar to our samples: Pd/TiO_2_/Pd), the formation of a conductive filament (CF) by oxygen vacancies is considered the main mechanism for electroforming.^34^ Once electroformed, resistance can be switched by rupturing and reforming the CFs.^33,35^ Correlating the change in the properties of the memristor layer to modifications in RS properties can help us gain insights into the underlying switching mechanism and pave the way for optimizing memristor performance for various real-world applications.

The properties of MIM structures can be modified by changing parameters such as thickness, surface roughness, composition and preparation method. TiO_2+*x*_ (oxygen overstoichiometric) thin films, prepared in excess oxygen during sputter deposition, are reported to exhibit electroforming-free RS and modified properties such as resistance ratio.^36^ Post-synthetic treatments, such as annealing and introducing defects or impurities, can also modify the structural properties. In recent years, several studies have been conducted to induce or modify RS using ion implantation or irradiation in MIM structures, as ion beams enable the controlled introduction of dopants, impurities, and defects.^37–39^ Irradiating oxide-based memristors with inert ions such as argon (Ar) optimizes properties such as electroforming and device-to-device variability of memristors.^37,39,40^ Doping with metal ions such as Co has also been reported to improve RS properties, such as the resistance ratio.^41^ However, there remains a gap in understanding how different ions, with varying reactivity, influence the underlying microstructure and switching behaviors. In the present paper, we elaborate on the microstructural and chemical modifications resulting from the implantation with two light ions with comparable mass and penetration depth but different chemical activity, *viz.*, neon (^20^Ne) and oxygen (^16^O) ions. Both ^16^O and ^20^Ne ions produce similar collision cascades under the same implantation conditions, as simulated in SRIM-2013.^42^ ^16^O ions can replenish or redistribute oxygen in TiO_2_, possibly changing the local stoichiometry and saturating oxygen vacancies. A comparative study between ^16^O and ^20^Ne implanted samples can help to decouple switching properties attributed to ion-induced defects alone (in the case of Ne ions) and such defects combined with saturation of pre-existing vacancies and local changes in the stoichiometry of the metal oxide film (in the case of O ions). Introduction of different switching modes and modifications in switching properties, such as electroforming voltage, resistance ratio, stability of resistance states over time (retention), and switching cycles (endurance), are explored.

## 2 Experimental Section

Si ⟨100⟩ wafers, purchased from Siegert Wafer, were subjected to the RCA cleaning process to produce substrates of acceptable quality to grow memristor films. A (275 ± 5) nm thick SiO_2_ layer was grown on the Si wafer by thermal oxidation in a vertical furnace (Micro TF-6, Koyo Lindberg). The wafer was diced into 10 mm × 10 mm substrates on which the memristor structure (Pd/TiO_2_/Pd/Ti) was deposited at room temperature using a UHV-compatible magnetron sputtering system with a background pressure of 5 × 10^–8^ mbar or lower. A quartz crystal microbalance (QCM) was used in the system to estimate the deposition rates. During deposition, the sample stage was rotated at 10°/s for film uniformity. Table 1 compiles the details of the multilayered structure deposited by magnetron sputtering. Figure 1 gives the schematic illustration and electric measurement configuration of the samples. The adhesion layer (Ti) and bottom electrode (Pd-BE) were deposited on the whole substrate. An approximately 2 mm × 10 mm area was masked with Al foil while depositing TiO_x_. This masking was performed in order to have Pd-BE available for electrical contact. Four top electrodes (Pd-TE), each measuring approximately 2 mm × 2 mm, were deposited on top of TiO_x_ while masking the rest of the sample.

**Figure 1:**
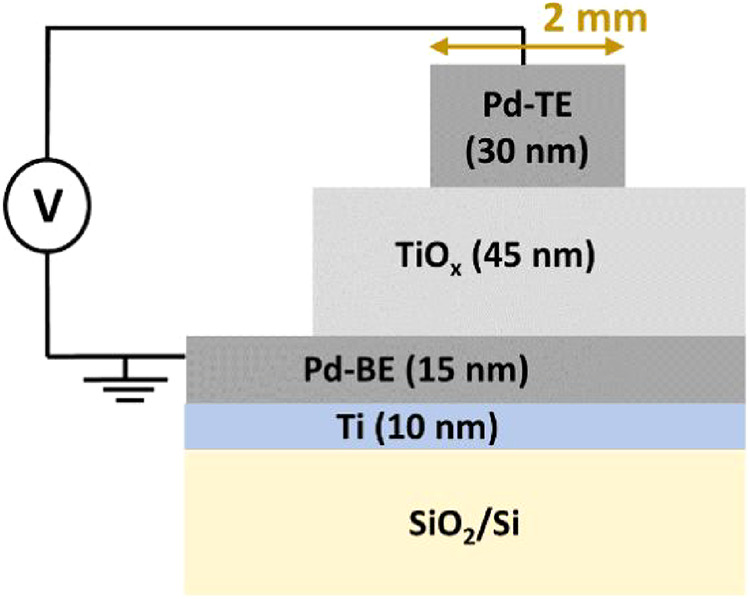
Schematic illustration and electrical measurement configuration of Pd/TiO_*x*_/Pd/Ti/SiO_2_/Si structures. The lateral dimension of the sample is in millimeters (mm), whereas the longitudinal dimension (film thickness) is in nanometers (nm).

**Table 1:** Details of Layers of the Sample and Deposition Conditions Used in Magnetron Sputtering^a^

Layer	Purpose	*t* [nm]	*P* [W]	*F* (Ar) [sccm]	*F* (O_2_) [sccm]	*p* [10^–3^ mbar]	Deposition Rate [nm/min]
Ti	Adhesion	10	150	10		5.6	5.0 ± 0.5
Pd	Bottom Electrode (BE)	15	50	10		5.6	14.5 ± 0.5
TiO_x_	Insulating layer	∼45	150	20	2	6.1	5.5 ± 0.5
Pd	Top Electrode (TE)	30	50	10		5.6	14.5 ± 0.5

a “*t*” is the thickness of the deposited film, “*P*” is the discharge power of the magnetron, “*F* (Ar)” and “*F* (O_2_)” give the flow rate of Ar and O_2_ gas, respectively, while depositing different layers and “*p*” is the pressure in the sputtering chamber during deposition.

Stoichiometry of reactively sputtered and ion-implanted TiO_*x*_ was measured using Time of Flight Elastic Recoil Detection Analysis (ToF-ERDA) at the Tandem Laboratory, Uppsala University.^43^ Measurements were done with a primary beam of 36 MeV ^127^I^8+^ ions, with both incident and detection angles of 67.5° with respect to the sample surface normal. The composition depth profiles from ToF-ERDA measurements were calculated using Potku.^44^ Rutherford Backscattering Spectrometry (RBS) was performed using a beam of 2 MeV ^4^He^1+^ ions, with a detector placed at a backscattering angle of 170°, to measure the areal density of the TiO_*x*_ layer. The mass density of the TiO_2_ layer was calculated to be 3.6 g cm^–3^ using the areal density from RBS and the layer thickness from Transmission Electron Microscopy (TEM). The TiO_*x*_ layer was implanted at room temperature under normal incidence with ^16^O^1+^ and ^20^Ne^1+^ ions, both at an energy of 8 keV. The energy was chosen for ^16^O^1+^ ions to create an oxygen-rich layer in the TiO_2_ layer, without affecting the electrodes. The same energy was used for ^20^Ne^1+^ ions to induce similar collision cascades to compare the results. All implantations were performed using a 350 kV ion implanter at the Tandem Laboratory, Uppsala University. Table 2 compiles a complete list of the ions, energy (*E*), range (*R*_P_) and longitudinal straggling (σ_long_) of the projectiles in the TiO_2_ layer, ion fluence, and the expected peak increase in oxygen content over stoichiometric TiO_2_ if all implanted oxygen were retained. *R*_P_, σ_long_ and the expected increase in oxygen content with implantation were calculated using SRIM-2013,^42^ with a mass density of 3.6 g cm^–3^ for the TiO_2_ layer.

**Table 2:** List of the Projectiles Implanted in the TiO_2_ Layer (Implantations Were Done before Depositing the Top Pd Electrode) with Their Kinetic Energy (*E*_K_), Range, Longitudinal Straggling (σ_long_) and Ion Fluence^a^

Ion	*E*_K_ [keV]	Range [nm]	σ_long_ [nm]	Ion Fluence [ions/cm^2^]	Expected Peak Implanted O [%]
^16^O	8	17	8	1.17 × 10^16^	10
				2.93 × 10^16^	25
^20^Ne	8	14	7	1.17 × 10^16^	
				2.93 × 10^16^	

a *R*_p_ and straggling were calculated using SRIM-2013.^42^ The rightmost column gives the expected peak increase in oxygen content over stoichiometric TiO_2_ if all implanted O were retained. The retention of implanted O is discussed further in Section 3 below.

The cross-section of the samples was studied using Transmission Electron Microscopy (TEM). TEM lamellae were cut out of the samples by a Zeiss FIB/SEM Crossbeam 550 with a Ga Ion-Sculptor gun, and analysis was conducted with a FEI Titan Themis 200 system at an acceleration voltage of 200 kV. A layer of Pt, initially using an electron gun and then with a Ga ion gun, was coated over the samples before cutting out the lamella to prevent the region of interest below the Pt layers from getting damaged during milling. TEM image processing was performed using ImageJ.^45^ Surface characterization was carried out using Atomic Force Microscopy (AFM) in contact mode using PSIA XE150 AFM, and image processing was performed using Gwyddion.^46^

X-ray Photoelectron Spectroscopy (XPS) and Hard X-ray Photoelectron Spectroscopy (HAXPES) were used to characterize oxygen vacancies in as-prepared and implanted samples by studying the change in ratio of Ti^3+^ and Ti^4+^ oxidation states. An Al Kα (1487 eV) source was used for XPS to probe depths of 2–5 nm; whereas a liquid Ga Kα (9252 eV) source was used for HAXPES to probe depths of 10–20 nm. The TiO_2_ layer was coated with a 2 nm Pd capping layer to avoid oxidation of the layer. The measurements were performed at the Kai Siegbahn Laboratory, Uppsala University, using a Scienta Omicron HAXPES lab equipment and an EW4000 electron analyzer. The experimental data were fitted using pseudo-voigt functions and a Shirley-type background.

Electrical characterization was performed by applying a voltage to the top electrode (TE) and grounding the bottom electrode (BE) using a Keysight B1500A Semiconductor Device Parameter Analyzer. In the present work, one switching cycle refers to two applied voltage sweeps, one during which the voltage is positive and the other during which it is negative. For every voltage sweep, a maximum allowed current, referred to as compliance current (*I*_cc_), was specified. If a current *I* ≥ *I*_cc_ was measured, the actual applied voltage was limited by the instrument to the voltage value for which *I* = *I*_cc._ Retention or stability of the resistance state over time was studied by applying a constant voltage of 5 mV across the sample and measuring current over time.

## 3 Results and Discussion

The as-prepared memristive film has a stoichiometric composition corresponding to TiO_2_, with an oxygen atomic fraction of (67 ± 1)%, as verified by ToF-ERDA. The stoichiometry modification of the TiO_2_ film resulting from ion implantation is limited to being within the statistical error on the ToF-ERDA measurement (less than ± 1.5% atomic fraction). In the case of ^16^O implantation, the measured O/Ti ratio is lower than the expected ratios in Table 2, indicating that ion-induced release of O during implantation leads to an equilibrium ^16^O concentration close to that of stoichiometric TiO_2_ (Figure S1 in the Supporting Information). Figure 2 shows (a) the TEM image for the cross-section of the as-prepared sample; and the scanning transmission electron microscopy (STEM) images for the cross-section of the sample implanted with (b) ^20^Ne and (c) ^16^O ions at a fluence of 2.93 × 10^16^ ions cm^–2^. The expected concentration profile of the implanted ions in the TiO_2_ layer, simulated using SRIM-2013, is plotted as yellow lines in Figure 2b and 2c, with the left edge of the image corresponding to zero concentration. The as-deposited and all implanted TiO_2_ films exhibit an amorphous structure as indicated by the fast Fourier transform (FFT) of the high-resolution TEM images of the TiO_2_ layer in Figure S2­(a–c). In the Ne-implanted samples, a bright-colored sublayer is observed in the STEM image (Figure 2b), at depths of (5–20) nm in the TiO_2_ layer. This contrast corresponds to the presence of Ne atoms implanted in the TiO_2_ layer (refer to Figure S2­(d–f) for Energy Dispersive X-ray Spectroscopy (EDS) measurements) and coincides with the SRIM-simulated implantation profile plotted in Figure 2b. In O-implanted samples, bright-colored regions are observed in the STEM image, beginning at a depth of (15 ± 2) nm in the TiO_2_ layer. EDS measurements show no measurable change in oxygen profile across the TiO_2_ layer after ^16^O implantation (Figure S2­(g)); however, the bright spots in the STEM images indicate the presence of local under-dense regions, suggesting local structural modifications. A smoother interface between the TiO_2_ film and Pd-TE is observed after ion implantation. As the ion-implantation is performed before depositing Pd-TE, the incoming ions modify the surface of the TiO_2_ layer.^47^ Atomic Force Microscopy (AFM) measurements confirm that the root-mean-square (RMS) roughness of the TiO_2_ surface, and thus the TE­(Pd)-TiO_2_ interface roughness, decreases with ion implantation (refer to Figure S3). A similar reduction in RMS roughness is observed for all ion implantation conditions. The effect of 8 keV ions on the BE­(Pd)-TiO_2_ interface roughness is negligible, as the fraction of ions reaching the BE is an insignificantly small percentage of incident ions (0% and 0.005% of the incident Ne and O ions, respectively, as simulated in SRIM-2013).

**Figure 2:**
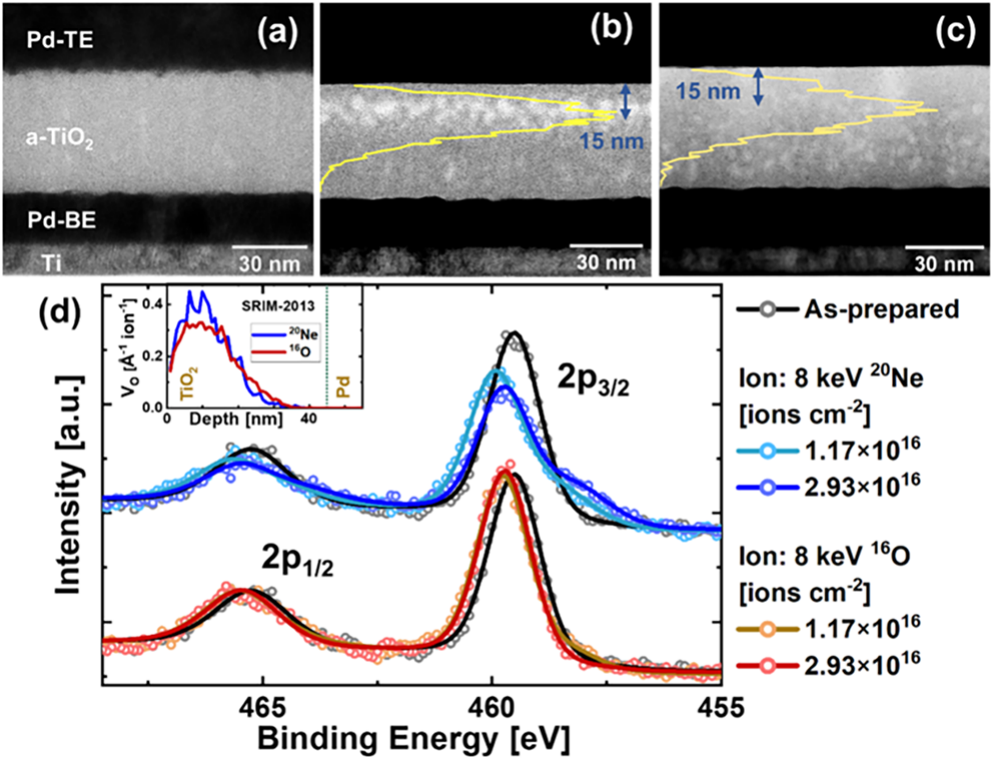
(a) TEM image of the as-prepared sample; STEM image of the sample implanted with (b) ^20^Ne and (c) ^16^O ions at a fluence of 2.93 × 10^16^ ions cm^–2^. The yellow lines indicate the SRIM-2013 simulated concentration profiles of (b) Ne and (c) O ions implanted in the samples; (d) HAXPES Ti 2p core level spectra and curve fits for as-prepared and implanted samples. Hollow circles represent the experimental data, and solid lines are the sum of the fitted curves. Inset: Oxygen vacancy concentration profiles for ^20^Ne and ^16^O ions, simulated using SRIM-2013, for comparison with the HAXPES information depth of 10–20 nm.

To gain further insight into the ion-induced modifications, XPS and HAXPES analysis were employed. From the SRIM-2013 simulation (inset in Figure 2d), ion implantation is expected to induce oxygen vacancies in the initial layers of the TiO_2_ layer with the peak vacancy concentration at a depth of 6–15 nm. The Ti 2p core level spectra, measured with Al Kα, for the implanted samples are quite similar to those for the as-prepared sample, indicating no conclusive results regarding changes in vacancy concentration in the initial 2–5 nm range. Figure 2d shows the HAXPES spectra and curve fits for as-prepared and implanted samples for Ti 2p core level measured with Ga Kα, which show more bulk-sensitive information at a depth of 10–20 nm (refer to Figure S4 for HAXPES Ti 2p core level spectrum for the as-prepared sample along with the curve fits for all the peaks, the sum of the fitted curves and the background). The peak at ∼459.5 eV for Ti 2p_3/2_ and the peak at ∼465 eV for Ti 2p_1/2_ correspond well with Ti^4+^. The binding energy of Ti^3+^ is approximately 1.5 eV lower and observed as a small shoulder on the lower binding energy side of the main peak. It is clearly observed for samples implanted with Ne ions for the Ti 2p_3/2_ peak. Fitting the experimental spectra, the relative amount of Ti^3+^ is calculated for the Ti 2p_3/2_ core level peak, which is the ratio of the area under the Ti^3+^ peak to the total area under the Ti 2p_3/2_ peak. The as-prepared sample is calculated to have 3% Ti^3+^, which increases to 28% and 31% Ti^3+^ after Ne implantation at fluences of 1.17 × 10^16^ and 2.93 × 10^16^ ions cm^–2^, indicating an increase in oxygen vacancy concentration. In the case of O implantation, oxygen ions are implanted in the TiO_2_ film in addition to the formation of oxygen vacancies. As a result, no significant change in HAXPES spectra is observed, and a 3–10% Ti^3+^ concentration is calculated in the O-implanted samples, which is similar to the as-prepared sample.

Overall, ion implantation leads to structural and local compositional changes in the insulating layer, i.e., TiO_2_, which can influence the switching properties of the Pd/TiO_2_/Pd memristors as discussed below. The as-prepared Pd/TiO_2_/Pd samples require an initial electroforming step before the resistance can be switched by changing the polarity of the applied voltage.^35^ The voltage at which a sudden increase in current is observed (digital switching), indicating the formation of stable conductive filaments (CFs) of oxygen vacancies (V_o_^••^) between the electrodes, is referred to as electroforming voltage (*V*_Ef_). The as-prepared samples have an initial resistance of (4 ± 1.5) MΩ and *V*_Ef_ of (7.5 ± 1.5) V. All the categories of ion-implanted samples are observed to electroform into a relatively lower resistance state than the initial resistance, presumably due to the formation of CFs of V_o_^••^ between the top and bottom electrodes (refer to Figure S5 for electroforming steps). Figure 3 plots (a) initial resistance and (b) *V*_Ef_ for as-prepared and implanted samples as a function of ion fluences, and (c) gives a scatter plot of the initial resistance and corresponding *V*_Ef_ for all the as-prepared and implanted samples. Ne-implantation has a more prominent and distinct effect on both initial resistance and V_Ef_ than O-implantation. Both initial resistance and *V*_Ef_ decrease with Ne implantation. These changes can be attributed to additional vacancies in the upper part of the TiO_2_ film being introduced after Ne-implantation, leading to a reservoir of V_o_^••^ in the upper part of the film, as concluded from the HAXPES spectra. The increase in the concentration of V_o_^••^ reduces the resistance of the sample and facilitates the formation of CFs, thus reducing *V*_Ef_. A decrease in interface roughness between the electrode and the memristor layer has been reported to increase *V*_Ef_.^22,24,48^ However, in the present case, the introduction of additional V_o_^••^ in Ne-implanted samples is observed to have a more pronounced effect on the *V*_Ef_ than the decrease in the TE-TiO_2_ interface roughness. In the case of O implantation, while the changes in the initial resistance of the samples are relatively small compared to Ne-implanted samples, an overall decrease in initial resistance is observed with an increase in O fluence. *V*_Ef_ shows a nonlinear trend characterized by an initial increase followed by a decrease as a function of O fluence. Although no significant change in V_o_^••^ concentration is observed from HAXPES spectra, a reduction in Pd­(TE)-TiO_2_ interface roughness and microstructural changes in the TiO_2_ film (STEM image in Figure 2c) are observed, which can affect the properties of the implanted samples.

**Figure 3:**
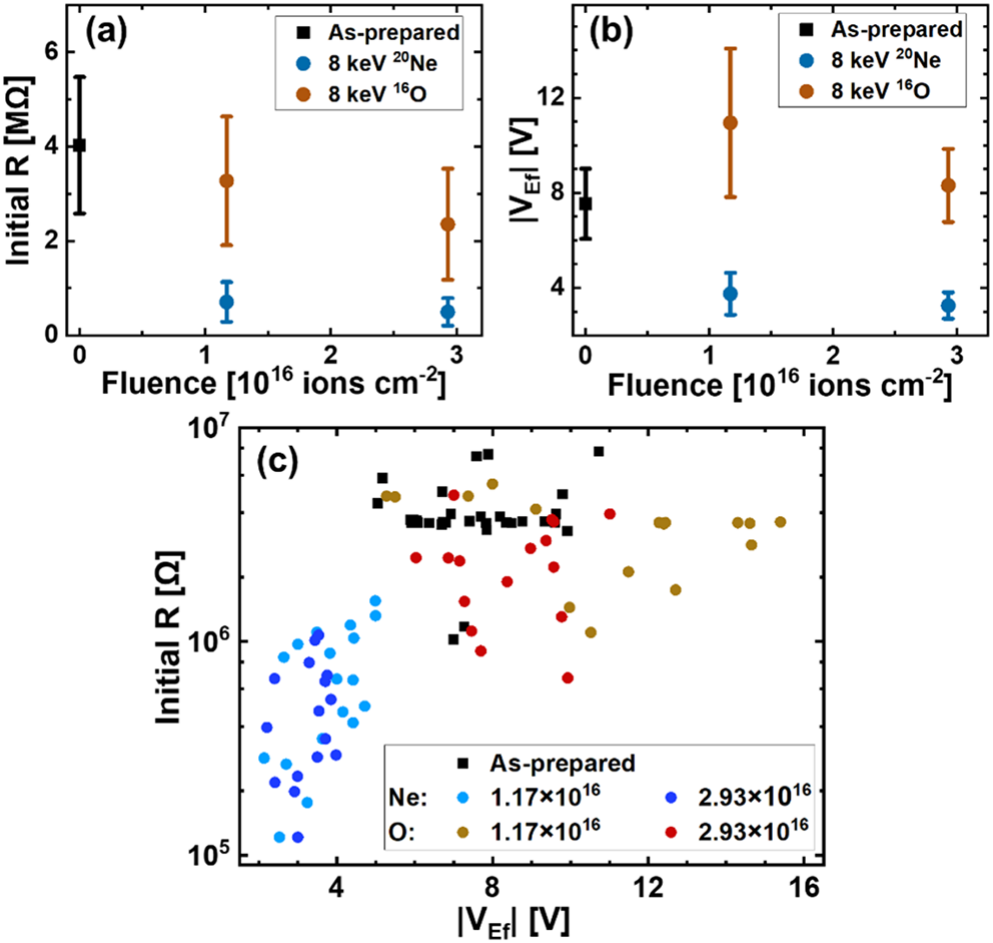
(a) Initial resistance of Pd/TiO_2_/Pd structure and (b) electroforming voltage (*V*_Ef_) as a function of ion fluence; (c) scatter plot of the initial resistance and corresponding *V*_Ef_ for all the as-prepared and implanted samples (fluence in terms of ions cm^–2^).

### 3.1 Electroforming-Free Resistive Switching—Short-Term Memory (STM)

When voltage sweeps from 0 V → *V*_max_ → 0 V are applied to the TE of the as-prepared sample, with alternating polarity and |*V*_max_| < *V*_Ef_, there is no significant change in resistance without the electroforming step. In contrast, the samples implanted with Ne ions (for both fluences) can be switched by changing the polarity of the voltage sweep. For O-implantation, no resistive switching is observed in samples with a fluence of 1.17 × 10^16^ ions cm^–2^; when the fluence is increased to 2.93 × 10^16^ ions cm^–2^, two distinct resistance states are observed. Figure 4a shows the resistance of the as-prepared sample and implanted samples over multiple switching cycles for |*V*_max_| < *V*_Ef_. For samples that do not exhibit resistive switching, the resistance measured before and after the voltage sweep is referred to as resistance state 1 (RS1) and resistance state 2 (RS2), respectively. The distinct resistance states are referred to as high resistance state (HRS) and low resistance state (LRS). The reproducibility or endurance of HRS and LRS over 100 switching cycles (switched at *I*_cc_ = 0.1 mA) is shown in Figure 4a. The switching occurs between two comparatively lower-resistance states in the Ne-implanted samples, as the initial resistance of these samples is lowered due to an increase in V_o_^••^ concentration. The stability of a given resistance state (i.e., retentivity) is assessed by applying a constant 5 mV voltage to TE and measuring the current over time. The corresponding resistance $\left(R\;\left[\Omega \right]=\frac{5\times 10^{-3}\;\left[V\right]}{I\;\left[A\right]}\right)$ is calculated and plotted in Figure 4b, which shows the retentivity of both LRS and HRS for the 50th switching cycle for both Ne-implanted samples and for the 68th switching cycle for the O-implanted sample. The resistance states are observed to be unstable and relax toward the initial resistance over time, i.e., the memory is volatile or short-term. Figure 4 shows the typical IV curves before electroforming, at *I*_cc_ = 0.1 mA, for samples implanted with 8 keV ^20^Ne^1+^ at fluences of (c) 1.17 × 10^16^ and (d) 2.93 × 10^16^ ions cm^–2^; and (e) 8 keV ^16^O^1+^ at a fluence of 2.93 × 10^16^ ions cm^–2^. These IV curves correspond to HRS and LRS in Figure 4a and exhibit analog bipolar switching behavior where the sample is set to LRS during the positive sweep and reset to HRS during the negative sweep. For switching at *I*_cc_ = 0.1 mA, O-implanted samples require voltage sweeps with |*V*_max_| = 6 ± 1 V, whereas only |*V*_max_| = 2 ± 0.5 V is required for Ne-implanted samples. If *I*_cc_ is reduced, the Ne-implanted samples can be switched at a lower voltage, but it also reduces the resistance ratio (*R*_Ratio_ = *R*_HRS_/*R*_LRS_) as the LRS increases. The difference in RS at *I*_cc_ = 0.01 mA, observed in Ne-implanted samples with different fluences, can be attributed to the difference in their V_o_^••^ concentration. Figure 4f compares the endurance of HRS and LRS over 100 switching cycles for Ne-implanted samples at both fluences, switched at *I*_cc_ = 0.1 and 0.01 mA. The corresponding IV curves for switching at *I*_cc_ = 0.01 mA, for samples implanted with 8 keV ^20^Ne^1+^ at fluences of (g) 1.17 × 10^16^ and (h) 2.93 × 10^16^ ions cm^–2^ are also shown in Figure 4. About 14 samples for each of the three implantation conditions exhibiting switching without electroforming were tested, and all tested samples showed electroforming-free RS. There is a device-to-device variation in absolute resistance states, as the initial resistance varies across the ion-implanted samples (as observed in Figure 3c).

**Figure 4:**
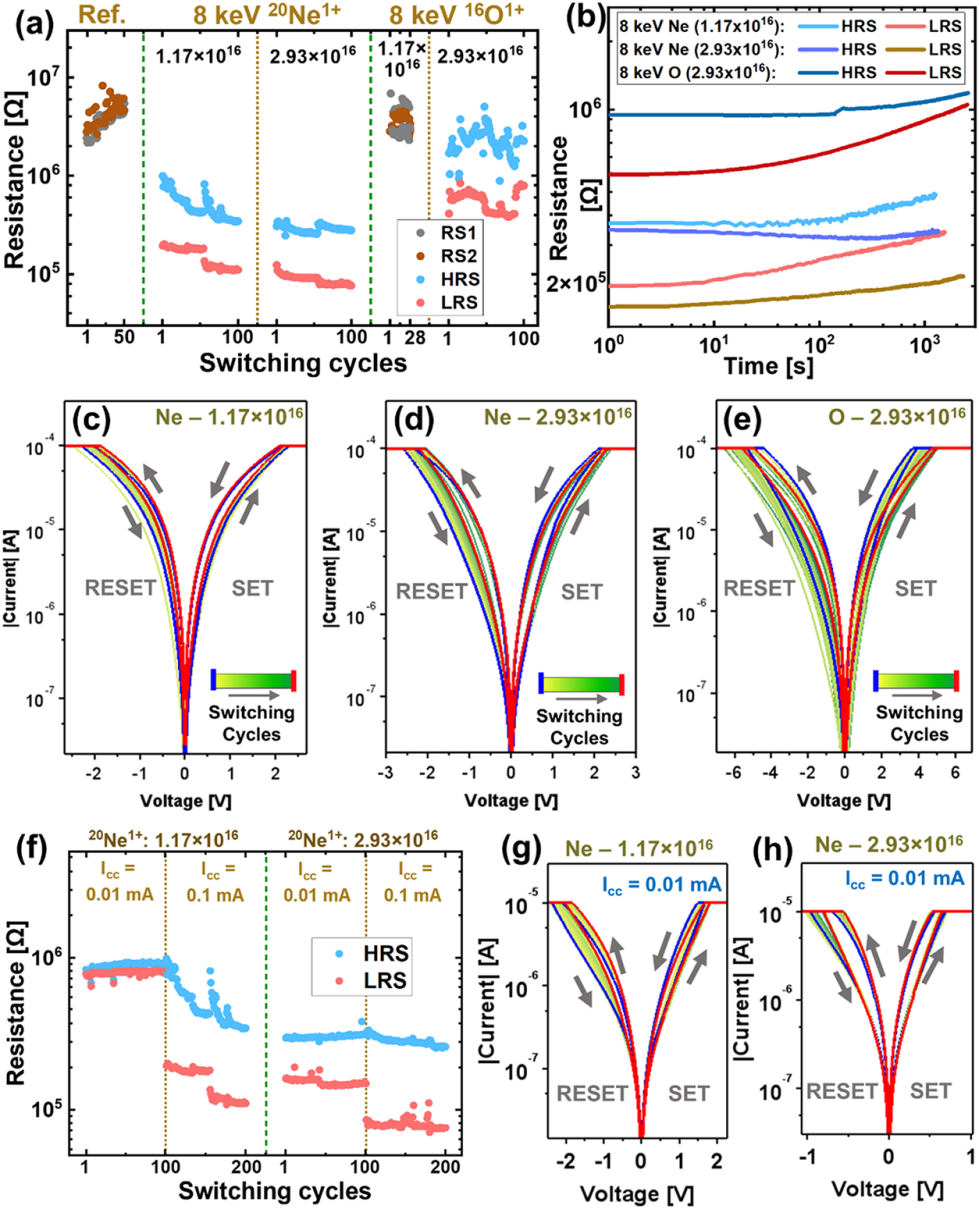
(a) Comparison of electroforming-free RS (at *I*_cc_ = 0.1 mA) in the implanted samples to the as-prepared (or Ref.) sample in terms of endurance; (b) retentivity of HRS and LRS for samples that exhibit electroforming-free RS; IV curves showing electroforming-free RS (at *I*_cc_ = 0.1 mA) in the samples implanted with 8 keV ^20^Ne^1+^ ions at fluences of (c) 1.17 × 10^16^ and (d) 2.93 × 10^16^ ions cm^–2^ and (e) 8 keV ^16^O^1+^ ions at a fluence of 2.93 × 10^16^ ions cm^–2^; (f) comparison of endurance of Ne-implanted samples for switching at different *I*_cc_; IV curves showing electroforming-free RS (at *I*_cc_ = 0.01 mA) in the samples implanted with 8 keV ^20^Ne^1+^ ions at fluences of (g) 1.17 × 10^16^ and (h) 2.93 × 10^16^ ions cm^–2^.

The electroforming-free analog RS observed in Ne-implanted samples can be explained by the migration of V_o_^••^ in the TiO_2_ film introduced by implantation, which is observed in HAXPES spectra. The drift and diffusion of V_o_^••^ can be described by the simple 1D ion model by Mott and Gurney.^49^ Once the external voltage is removed, the forces driving the *V*_*o*_^••^ disappear, and the system relaxes. Thus, the resistance states are not stable after the voltage across the sample is removed, and plots in Figure 4b can be fitted to calculate the diffusion time constant (τ) for V_o_^••^, and subsequently, correlate the diffusion and drift velocities at different applied voltages. The LRS state for a sample implanted with Ne ions at a fluence of 1.17 × 10^16^ ions cm^–2^, for example, was fitted with a biexponential function: $R\left(t\right)=R_{0}+R_{1}e^{-\left(t/\tau_{1}\right)}+R_{2}e^{-\left(t/\tau_{1}\right)}$ , with τ_1_ = 60 s and τ_2_ = 1083 s. Two characteristic τ values indicate a faster vacancy redistribution and a slower bulk vacancy diffusion. Diffusivity (*D*) of 9 × 10^–15^ cm^2^ s^–1^ is calculated for τ_2_ = 1083 s across the thickness of the TiO_2_ layer (*L* = 45 nm) using the expression $D=\frac{L^{2}}{\tau }$ . This value is higher than the reported values for crystalline TiO_2_,^47^ but is expected, as our samples are amorphous films with further increase in V_o_^••^ concentration by implantation. It is important to note that although the resistance was measured at a voltage of 5 mV, $\frac{\mathrm{qV}}{2k_{B}T}=0.19<1$ at room temperature (298 K), indicating that the thermal motion dominates the motion of V_o_^••^. When voltages above 0.3 V are applied across the sample, $\frac{\mathrm{qV}}{2k_{B}T}>10$ , indicating that the drift dominates the *V*_o_^••^ migration over diffusion and thus, resistive switching. For example, using the *D* calculated above and an applied voltage of 1 V, the drift and the average diffusion velocity $\left(\frac{2D}{L}\right)$ ^50^ are 1.6 and 0.04 nm s^–1^, respectively. The drift velocity was calculated using the following expression (*E* = electric field; *a* = effective hopping distance = (oxygen number density)^−1/3^)^51^

$$v_{\mathrm{drift}}=\frac{2D}{a}\sinh\left(\frac{\mathrm{aqE}}{2k_{B}T}\right)$$

Figure 5 shows a schematic of the (a) as-prepared and (b) Ne-implanted sample, with V_o_^••^ indicated by olive green circles. The current flowing through the sample consists primarily of two contributions: electronic conduction and vacancy drift conduction, both influenced by the concentration and migration of V_o_^••^ in the film.^52^ When an external voltage is applied across the sample, the V_o_^••^ are formed at the +ve electrode, drift toward the −ve electrode, and are annihilated at the −ve electrode. Both the rates of vacancy formation and annihilation, as well as the drift velocity, depend on the applied voltage and the V_o_^••^ concentration profile.^35,53,54^ Thereby, when a lower external voltage is applied (when the *I*_cc_ is reduced), the resistance states change as seen in Figure 4f; and when the V_o_^••^ concentration profile is modified by fluence of Ne-implantation, the RS changes as observed distinctly at *I*_cc_ = 0.01 mA (Figure 4f). V_o_^••^ annihilated at the −ve electrode are indicated by red circles, whereas V_o_^••^ formed at the +ve electrode are indicated by green circles in Figure 5.

- When a voltage sweep from 0 V → −3 V → 0 V is applied, the pre-existing V_o_^••^ near the Pd-TE (-ve electrode), are presumably annihilated and additional V_o_^••^ are expected to form at the Pd-BE (+ve electrode). The presence of a reservoir of V_o_^••^ near the Pd-TE results in a higher rate of V_o_^••^ annihilation at Pd-TE than the rate of V_o_^••^ formation at the Pd-BE, i.e., a decrease in V_o_^••^ concentration in the memristor layer when −ve voltage is applied. The V_o_^••^ drift toward the Pd-TE, leading to an accumulation of the V_o_^••^ in a confined region near the Pd-TE interface. As a result, high conduction (V_o_^••^ rich) region is restricted to a localized spatial region close to the TiO_2_ and Pd-TE interface. Thus, the narrowing of the V_o_^••^ concentration profile, along with an overall decrease in V_o_^••^ concentration, results in an increase in the sample resistance when -ve voltage is applied to Pd-TE (Figure 5c).
- When a voltage sweep from 0 V → 3 V → 0 V is applied, V_o_^••^ in the reservoir near the Pd-TE (+ve electrode) drift toward the Pd-BE (-ve electrode), distributing the V_o_^••^ concentration into a wider region along the depth of the memristor layer and thus, increasing the high conduction (V_o_^••^ rich) spatial region in the TiO_2_ layer. Additional V_o_^••^ are formed at the Pd-TE and any pre-existing V_o_^••^ near the Pd-BE are annihilated. The combined effect of the widening of the V_o_^••^ concentration profile and a comparatively higher or similar formation rate than the annihilation rate result in a decrease in the sample resistance when a +ve voltage is applied to Pd-TE (Figure 5d).

**Figure 5:**
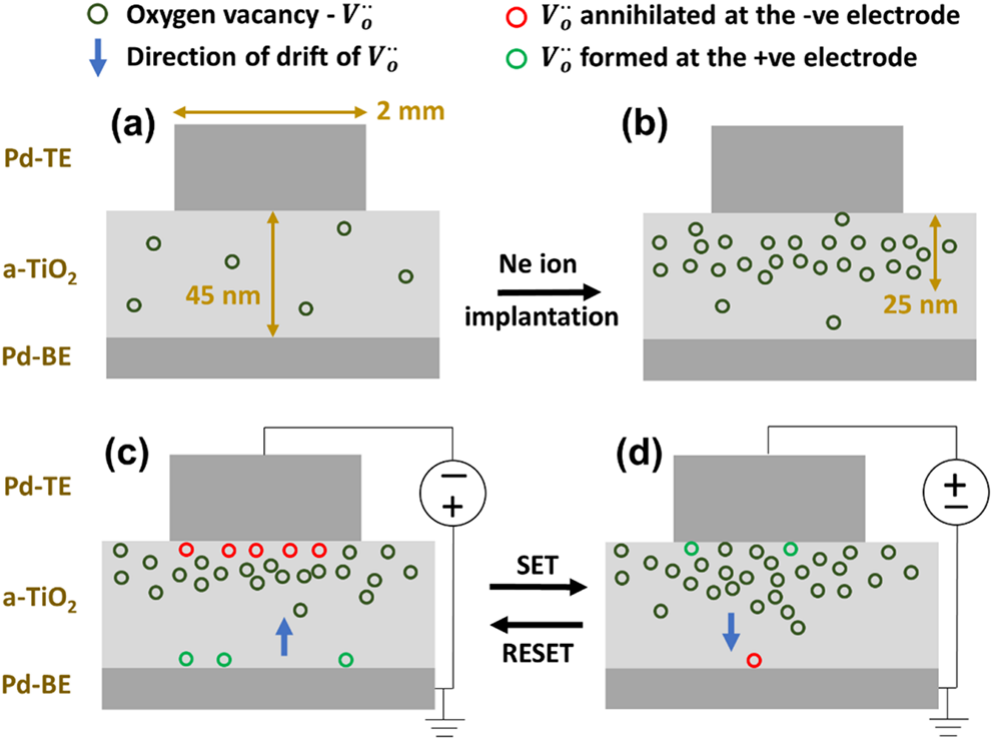
Schematics of (a) the as-prepared Pd/TiO_2_/Pd sample and (b) Ne-implanted sample, with oxygen vacancies (V_o_^••^) indicated by olive green circles. Schematics for the implanted sample, biased with (c) negative and (d) positive voltage at Pd-TE. Green circles indicate the V_o_^••^ formed at the +ve electrode, and red circles indicate V_o_^••^ annihilated at the -ve electrode. The blue arrow shows the direction of V_o_^••^ drift under the applied voltage.

The current–voltage conditions applied to the samples in this case are not enough to electroform or form stable CFs between the electrodes.

No electroforming-free RS is observed in samples implanted with ^16^O ions at a fluence of 1.17 × 10^16^ ions cm^–2^, but when the fluence is increased to 2.93 × 10^16^ ions cm^–2^, electroforming-free bipolar RS is observed. As discussed above, compared to the Ne-implanted samples, electroforming-free switching in O-implanted samples occurs between two higher resistance states and at higher voltages (|*V*_max_| = 6 ± 1 V). The low resistance states and the voltage required for switching are attributed to the formation of V_o_^••^ reservoirs in Ne-implanted samples. However, for the ^16^O-implanted samples, neither a significant increase in V_o_^••^ concentration in XPS and HAXPES spectra (Figure 2d), nor a distinct modification in the oxygen profile in ERDA (Figure S1) or EDS (Figure S2­(g)) is observed. However, structural changes, such as local under-dense regions, are observed in the STEM images of O-implanted samples (Figure 2c), suggesting that although the TiO_2_ films remain overall stoichiometric, ^16^O-implantation introduces local structural disorders that affect the vacancy formation and migration and contribute to electroforming-free RS. There is an interplay between the formation of V_o_^••^ and the implantation of incoming oxygen ions in the TiO_2_ film, which effectively maintains TiO_2_ stoichiometry despite continuous defect generation. Thus, the minimal overall chemical composition and detectable local structural changes can provide a consistent explanation for the observed electroforming-free RS observed in samples implanted with ^16^O ions at a fluence of 2.93 × 10^16^ ions cm^–2^.

### 3.2 Resistive Switching after Electroforming—Long-Term Memory (LTM)

Once stable CFs are formed in the electroforming step, the electronic conduction becomes the dominant mechanism for current flow in the sample. Thus, after electroforming, switching occurs between two comparably lower-resistance states (in the range of 10^3^ Ω) compared to the electroforming-free RS (in the range of 10^5^ Ω). Analog bipolar resistive switching is observed for *I*_cc_ ≥ 1 mA in the as-prepared and all the ion-implanted samples due to rupturing and reformation of CFs (refer to Figure S6 for typical IV curves after electroforming).^35^ Figure 6 shows the (a) reproducibility or endurance and (b) retentivity of HRS and LRS (obtained at *I*_cc_ = 1 mA) of the as-prepared samples and implanted samples after electroforming. Unlike the resistance states before electroforming, these resistance states are stable over time, i.e., the memory is nonvolatile or long-term. A total of 31 as-prepared samples and 14–16 samples for each implantation category were tested after electroforming. About 60% of the tested as-prepared samples and 40–45% of the implanted samples, tested for each implantation category, exhibited the typical RS behavior shown in Figure 6a.

**Figure 6:**
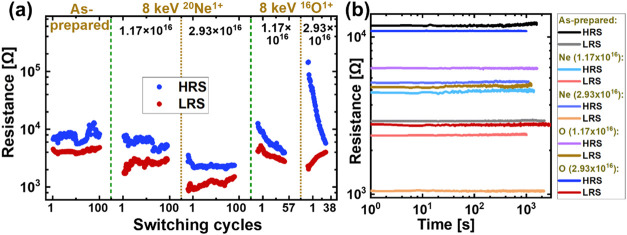
(a) Reproducibility or endurance and (b) retentivity of the LRS and HRS (*I*_cc_ = 1 mA) in as-prepared and implanted samples after electroforming. The fluence mentioned in the figures is in units of ions cm^–2^.

For as-prepared and Ne-implanted samples, the endurance for 100 switching cycles at a constant *I*_cc_ of 1 mA is shown in Figure 6a. The value of LRS in the initial switching cycles is lower for Ne-implanted samples than that for as-prepared samples. Ne implantation increases the V_o_^••^ concentration, which may promote the formation of thicker CFs, leading to a lower value of LRS.^28^ Additionally, these Ne-implanted samples are electroformed at a higher *I*_cc_ (1 mA) than the as-prepared samples (*I*_cc_ = 0.1 mA), which may affect the thickness of the CFs formed.^29^ The formation of these more robust/thicker filaments also makes it difficult to reset the sample after electroforming, resulting in ∼60% of Ne-implanted samples failing to reset to HRS, even when a higher current is allowed across the sample (for example, by increasing *I*_cc_ during the RESET step). The rest of the Ne-implanted samples that could be reset show an average endurance of 100 cycles at a constant *I*_cc_ of 1 mA, after which the *R*_Ratio_ deteriorates below 1.5. The as-prepared samples that were tested showed an average endurance of 200 switching cycles. In the case of O-implantation, only 57 and 38 switching cycles are plotted for samples implanted at fluences of 1.17 × 10^16^ and 2.93 × 10^16^ ions cm^–2^, respectively, in Figure 6a. This limited number of switching cycles is plotted because, when switched at a constant *I*_cc_ of 1 mA, the value of HRS decreased with each switching cycle, leading to deterioration in *R*_Ratio_ below 1.5 in less than 100 switching cycles. As observed in Figure 6a, the HRS for the first switching cycle in O-implanted samples increases with ion fluence. Consequently, the *R*_Ratio_ in the initial cycles (at *I*_cc_ = 1 mA) increases from ∼ 2 for the as-prepared samples to ∼6 and ∼50 for samples implanted at fluences of 1.17 × 10^16^ and 2.93 × 10^16^ oxygen ions cm^–2^, respectively (refer to Figure S7 for scatter plot of the *R*_Ratio_ in the initial cycles). However, the rate of HRS degradation with successive switching cycles also increases with increasing fluence. As a result of these competing effects, although the higher ^16^O fluence gives a higher *R*_Ratio_ in the initial cycles, the endurance over cycles decreases with increasing implantation fluence. For resistance states at *I*_cc_ = 1 mA, the samples implanted with ^16^O ions at fluences of 1.17 × 10^16^ and 2.93 × 10^16^ ions cm^–2^ show average endurance of 50 and 20 switching cycles, respectively.

Once the R_Ratio_ deteriorates (at a specific *I*_cc_, such as 1 mA), the *I*_cc_ was increased to test whether the samples could still switch between different resistance states. Increasing the *I*_cc_ reduces the value of resistance states for both the as-prepared sample and the implanted samples. Subsequently, the as-prepared samples can be switched between two lower resistance states by applying voltage sweeps at higher I_cc_. However, most Ne-implanted samples could not be RESET from this new LRS, even at *I*_cc_ as high as 100 mA. The samples implanted with O ions at a fluence of 1.17 × 10^16^ ions cm^–2^ could be reset by applying a voltage >5 V at a high *I*_cc_ of 100 mA. But after this reset, it was no longer possible to SET the samples to an LRS *via* voltage sweeps. Conversely, for the samples implanted with O ions at a fluence of 2.93 × 10^16^ ions cm^–2^, the initial HRS (i.e., HRS in the first switching cycle after electroforming) could be recovered by resetting the sample at a higher *I*_cc_. Figure 7a shows typical IV curves for samples implanted with 2.93 × 10^16^ oxygen ions cm^–2^, at a *I*_cc_ = 1 mA, where HRS decreases with each cycle while LRS remains comparably unchanged. When the voltage is swept from 0 V → 3 V → 0 V with *I*_cc_ increased to 4 mA, the sample resets to the initial HRS (red curve in Figure 7a). Figure 7b shows the recovery of HRS (or *R*_Ratio_) over multiple switching cycles, modulated by the *I*_cc_ for the RESET step along the positive voltage sweep. For a constant *I*_cc_ = 1 mA, the HRS exhibits continuous decay with each switching cycle. This decay is reversible and can be recovered at any stage by applying a higher *I*_cc_ (4 mA in this case), as shown on the left side of the dashed brown line in Figure 7b. A gradual change in the number of switching cycles over which HRS decays at a given *I*_cc_ indicates that the sample evolves under repeated switching. While extended endurance measurements are not carried out in the present study, the results demonstrate the recovery of HRS at higher *I*_cc_, confirming the underlying effect within the tested range. Alternatively, a stable and reproducible HRS can be maintained by resetting the sample at a higher *I*_cc_ (3 mA in this case, as compared to the *I*_cc_ = 1 mA used for setting the sample) in every switching cycle, as shown on the right side of the dashed brown line in Figure 7b. Figure 7c shows the retentivity of various LRS and HRS corresponding to different switching cycles in Figure 7b. All resistance states are stable over time, i.e., the memory is long-term (nonvolatile). Thus, by implanting ^16^O ions into the TiO_2_ layer, weighted and recoverable resistance states with LTM are introduced in the Pd/TiO_2_/Pd memristors. The recovery of HRS at higher current may be explained by Joule-heating-assisted vacancy diffusion^55,56^ in addition to field-driven vacancy drift. The rate of drift and formation/annihilation of V_o_^••^ depends on the voltage.^35^ As observed in Figure 7a, with each switching cycle, the voltage at which the sample reaches the *I*_cc_ reduces, thus reducing the maximum voltage applied across the sample, and thus, the field-driven vacancy drift. When the *I*_cc_ is increased, a higher voltage is allowed across the samples, resulting in a higher rate of drift and formation/annihilation of V_o_^••^. In addition, a higher current may generate more Joule heating,^57^ enhancing vacancy diffusion and rupturing of the CFs, resulting in resetting the sample to a higher value of HRS (or recovery of HRS).

**Figure 7:**
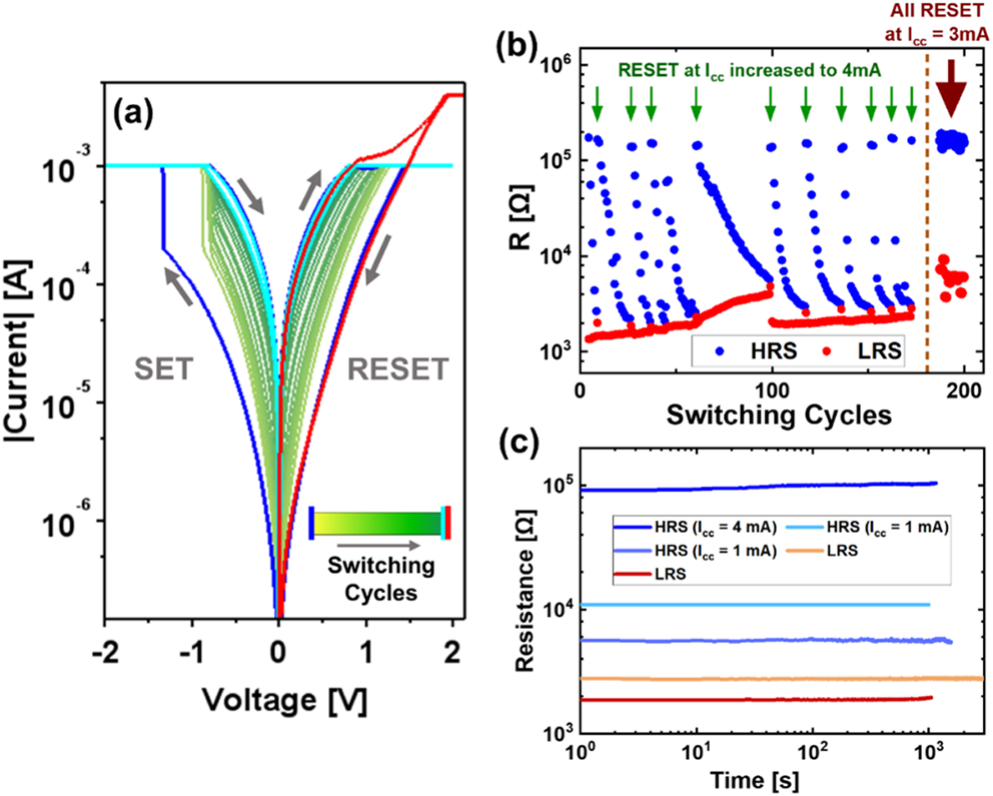
(a) IV curves, (b) endurance and (c) retentivity of weighted HRS and LRS for resistive switching observed in samples implanted with ^16^O ions at a fluence of 2.93 × 10^16^ ions cm^–2^. In (a), *I*_cc_ = 1 mA for the electrical measurements except for the red curve, where *I*_cc_ = 4 mA. The IV curves in (a) correspond to switching cycles from 9 to 26.5 in (b). In (b), on the left side of the brown dashed line—SET at *I*_cc_ = 1 mA and RESET at *I*_cc_ = 1 mA for all switching cycles except the ones marked with a green arrow, where the sample was RESET at *I*_cc_ = 4 mA; on the right side of the brown dashed line—SET at *I*_cc_ = 1 mA and RESET at *I*_cc_ = 3 mA for all cycles.

## 4 Conclusions

We have explored the modification of resistive switching (RS) in Pd/TiO_2_/Pd memristors by ion implantation. The as-prepared samples exhibit analog, bipolar, nonvolatile RS after electroforming. Ion implantation introduces controlled defects in the sample that could be used to improve or tune the properties of memristors for various applications. Oxygen (^16^O) and neon (^20^Ne) ions of 8 keV energy were implanted in the TiO_2_ layer. Table 3 compares the RS properties of as-prepared and all categories of implanted samples. The increase in V_o_^••^ by Ne-implantation, observed in HAXPES spectra (Figure 2d), introduces electroforming-free RS with short-term memory (STM) in the samples. It further reduces the electroforming voltage (*V*_Ef_), as the increase in the V_o_^••^ concentration facilitates the formation of CFs, and samples exhibit RS with long-term memory (LTM) after electroforming. In the case of O-implanted samples, the implanted oxygen also plays an active role in addition to the V_o_^••^ introduced by implantation. The ions introduce variation in the microstructure of the insulating layer (i.e., TiO_2_) while maintaining the overall stoichiometry. The *V*_Ef_ increases as the presence of additional oxygen within the insulating layer hinders the formation of CFs of V_o_^••^ connecting the two electrodes. While no further modification of composition is observed as the oxygen ion fluence increases from 1.17 × 10^16^ to 2.93 × 10^16^ ions cm^–2^, the reduction in V_Ef_ can be attributed to the continued ion-induced changes in the microstructure of the TiO_2_ layer. Additionally, electroforming-free RS with STM is observed in samples implanted with oxygen at a fluence of 2.93 × 10^16^ ions cm^–2^, but between two higher resistance states and at higher voltages (compared to Ne-implanted samples). Post-electroforming, all categories of the O-implanted samples exhibit bipolar resistive switching with LTM. The samples with higher fluence (i.e., 2.93 × 10^16^ ions cm^–2^) simultaneously exhibit LTM and weighted LTM with recoverable resistance states, which are modulated by the compliance current (*I*_cc_).

**Table 3:** Summary of Resistive Switching Properties in As-Prepared and Implanted Samples^a^

		8 keV ^20^Ne^1+^		8 keV ^16^O^1+^	
	As-prepared	1.17 × 10^16^ ions/cm^2^	2.93 × 10^16^ ions/cm^2^	1.17 × 10^16^ ions/cm^2^	2.93 × 10^16^ ions/cm^2^
Initial resistance (10^6^ Ω)	4 ± 1.5	0.7 ± 0.4	0.5 ± 0.3	3.3 ± 1.4	2.4 ± 1.2
RS before electroforming		STM	STM		STM
*V*_Ef_ (V)	7.5 ± 1.5	3.8 ± 0.9	3.3 ± 0.6	10 ± 3	8.3 ± 1.5
RS after electroforming	LTM	LTM	LTM	LTM	LTM + Weighted LTM with recoverable states

a All samples show analog bipolar resistive switching, both before and after electroforming

Memristors show significant promise for the future of neuromorphic computing, memory storage and in-memory computation, offering a pathway toward more efficient and brain-inspired electronic systems. We have demonstrated that defect engineering by ion implantation can be used to tune switching properties, ranging from the initial electroforming process to memory retention, and introduce different switching modes (electroforming-free RS with STM, RS with LTM, RS with weighted and recoverable resistance states with LTM). Extended cycling studies in samples implanted with oxygen at a fluence of 2.93 × 10^16^ ions cm^–2^ will help determine whether the decrease in the number of switching cycles over which HRS decays at a given *I*_cc_ (Figure 7b) is due to cycle-to-cycle variations or longer-term trends. Further in-depth experimental investigations are essential to fully understand the underlying mechanisms of electroforming-free RS and weighted long-term memory behavior with recoverable high-resistance states in O-implanted samples. A deeper understanding of these processes could enable the development of more reproducible and controllable resistive switching characteristics, ultimately advancing the reliability and functional tunability of memristor-based devices for practical applications.

## Supplementary Material



## Notes

Additional experimental data, including extended material characterization (ToF-ERDA, AFM, HAXPES, Energy Dispersive X-ray Spectroscopy (EDS) and HRTEM measurements) and electrical characterization (IV curves) for the as-prepared and ion-implanted Pd/TiO_2_/Pd memristors (PDF)
