# Supplementary material for: Tuning TiO2 Memristors by Defect Engineering: From Short-Term Memory to Recoverable Long-Term Resistance States
Source: ACS Appl Mater Interfaces. 2026 May 5;18(19):28009–19. doi: 10.1021/acsami.6c04844 (PMC13195577; doi:10.1021/acsami.6c04844)
Supplement: Supplementary file 1 [file am6c04844_si_001.pdf]

Supporting Information

Tuning TiO_2 memristors by defect engineering: From short-term memory to recoverable long-term resistance states

Rajdeep Kaur [a*], Tuan Thien Tran [a], Rebecka Lindblad [b], Zhen Zhang [c], Daniel Primetzhofer [a,d] and Petter Ström [a]

[a] *Division of Materials Physics, Department of Physics and Astronomy, Uppsala University, 751 20 Uppsala, Sweden*

[b] *Division of X-ray Photon Science, Programme of Condensed Matter Physics of Energy Materials, Department of Physics and Astronomy, Uppsala University, 751 20 Uppsala, Sweden*

[c] *Division of Solid-State Electronics, Department of Electrical Engineering, Uppsala University, 751 20 Uppsala, Sweden*

[d] *Tandem Laboratory, Uppsala University, 751 21 Uppsala, Sweden*

*Email: rajdeep.kaur@physics.uu.se

Time-of-Flight Elastic Recoil Detection Analysis (ToF-ERDA)

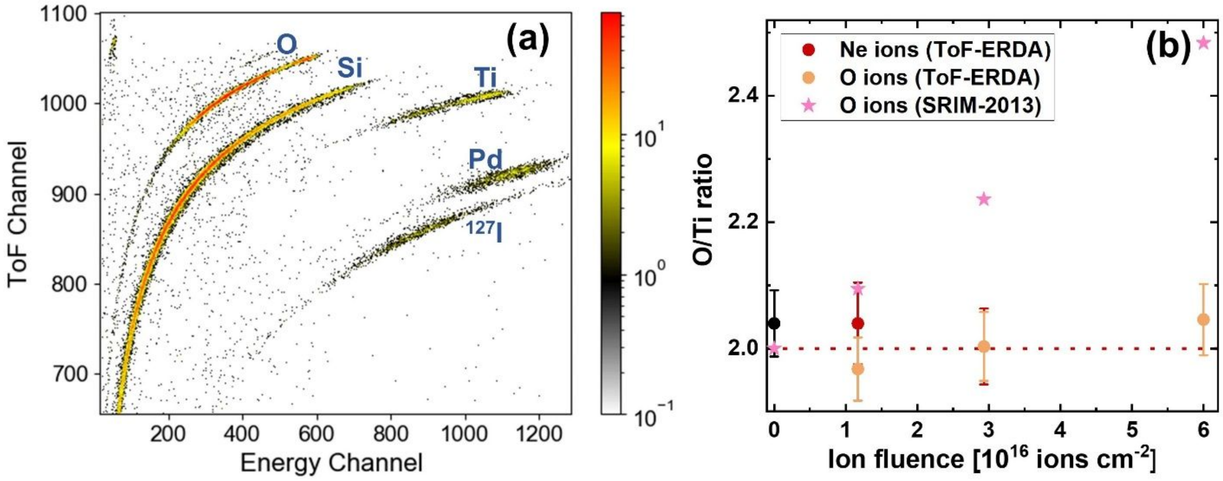

Figure S1. *(a) ToF-ERDA spectra for as-prepared samples (measured before depositing Pd-TE); (b) O/Ti ratio in TiO_x layer as a function of fluence of 8 keV $^{16}O^{1+}$ and $^{20}Ne^{1+}$ ions implanted in TiO_x. The pink stars in (b) give the expected O/Ti ratio in ^{16}O-implanted samples, calculated using SRIM-2013.*

Time-of-Flight Elastic Recoil Detector Analysis (ToF-ERDA) was performed to measure the compositional depth profiles of the ion-implanted samples. These measurements were performed before depositing the Pd top electrode (Pd-TE). **Figure S1** shows (a) the ToF-ERDA spectra for the as-prepared and (b) the stoichiometry, measured using ToF-ERDA, of the sample implanted with 8 keV $^{16}O^{1+}$ and $^{20}Ne^{1+}$ ions as a function of ion fluence. From SRIM-2013 calculations,[1] the O/Ti ratio is expected to increase linearly from 2 in as-prepared samples to ~2.24 in samples implanted with 2.93×10^{16} oxygen ions cm^{-2}, given that all the incoming oxygen ions were retained in the TiO_2 layer. As observed in Figure S1(b), within the measurement uncertainty, the O/Ti ratio or overall stoichiometry of the TiO_2 is unaffected by the implantation of oxygen ions. A plausible reason for the lack of change in oxygen composition is that incident ions induced the release of oxygen, leading to an equilibrium stoichiometry close to TiO_2.

Cross-section of the TiO_2 layer using Transmission Electron Microscopy (TEM):

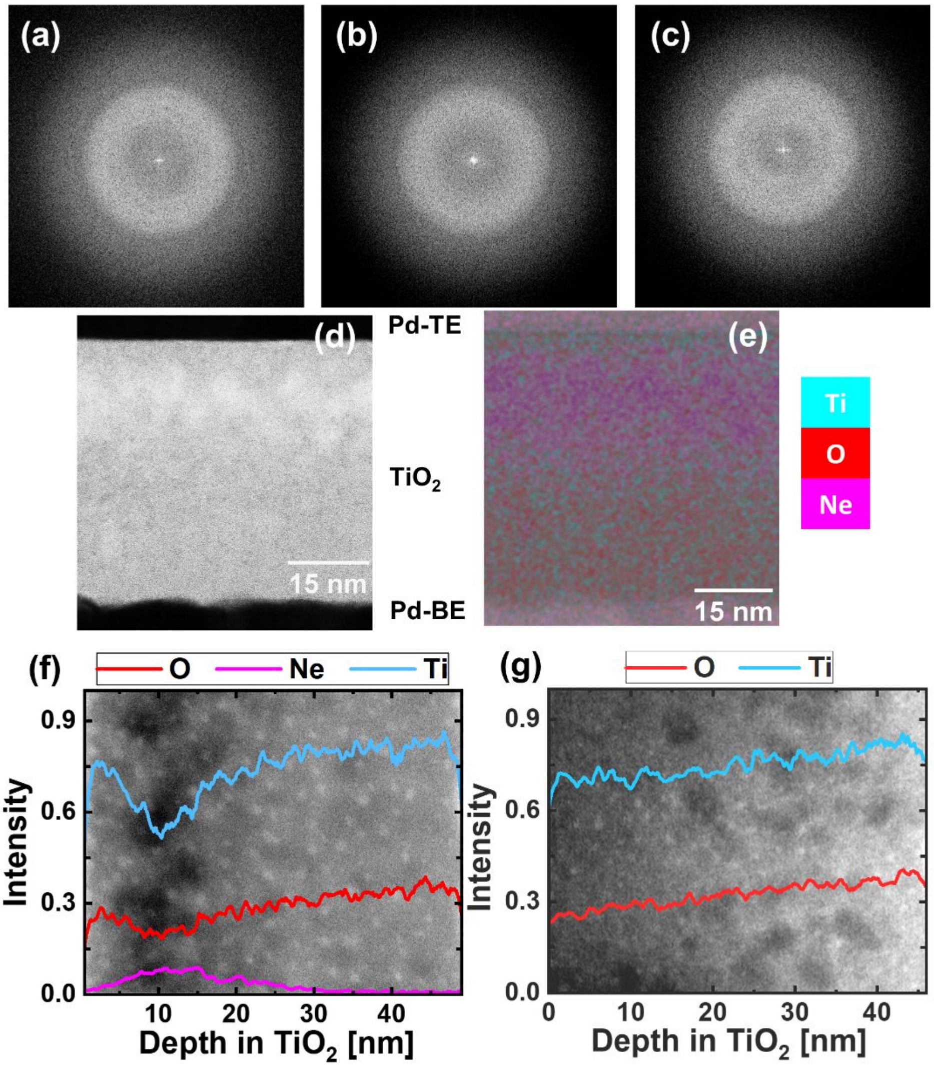

Figure S2. *Fast Fourier Transform (FFT) of the HRTEM images of TiO_2 layer in (a) the as-prepared sample and samples implanted with (b) ^{20}Ne and (c) ^{16}O ions at a fluence of 2.93×10^{16} ions cm^{-2}; (d) Bright field-STEM image and (e) the corresponding EDS colour map of the TiO_2 layer implanted with 8 keV $^{20}Ne^{1+}$ ions at a fluence of 2.93×10^{16} ions cm^{-2}; HAADF-STEM image along with the intensity of EDS signal across the depth of TiO_2 layer implanted with (f) ^{20}Ne and (g) ^{16}O ions at a fluence of 2.93×10^{16} ions cm^{-2}. The x-axis shows depth with respect to the Pd(TE)-TiO_2 interface.*

Figure S2 shows the fast fourier transform (FFT) of the high-resolution TEM (HRTEM) images of the TiO_2 layer in (a) the as-prepared sample and samples implanted with (b) ^{20}Ne and (c) ^{16}O ions at a fluence of 2.93×10^{16} ions cm^{-2}. The FFT indicates that the reactively sputtered TiO_2 film is amorphous and remains amorphous after implantation with ^{20}Ne and ^{16}O ions. Figure S2(d) shows the scanning transmission electron microscopy (STEM) image of the cross-section

of the TiO_2 layer implanted with 8 keV Ne ions at a fluence of 2.93×10^{16} ions cm^{-2}. A bright-coloured sublayer is observed in the STEM image, beginning at a depth of (6 ± 1) nm in the TiO_2 layer. The corresponding Energy Dispersive X-ray Spectroscopy (EDS) colour map in Figure S2(e) and the high-angle annular dark-field (HAADF) STEM image, along with the EDS signal across the depth of the TiO_2 layer in Figure S2(f), show that this bright sublayer corresponds to Ne atoms implanted in the TiO_2 layer. Figure S2(g) shows the HAADF-STEM image along with the EDS signal across the depth of the TiO_2 layer implanted with ^{16}O ions at a fluence of 2.93×10^{16} ions cm^{-2}. Although under-dense local regions are observed in the lower half of the TiO_2 layer implanted with ^{16}O ions, EDS measurements indicate no significant change in oxygen profile across the layer.

Surface characterisation using AFM:

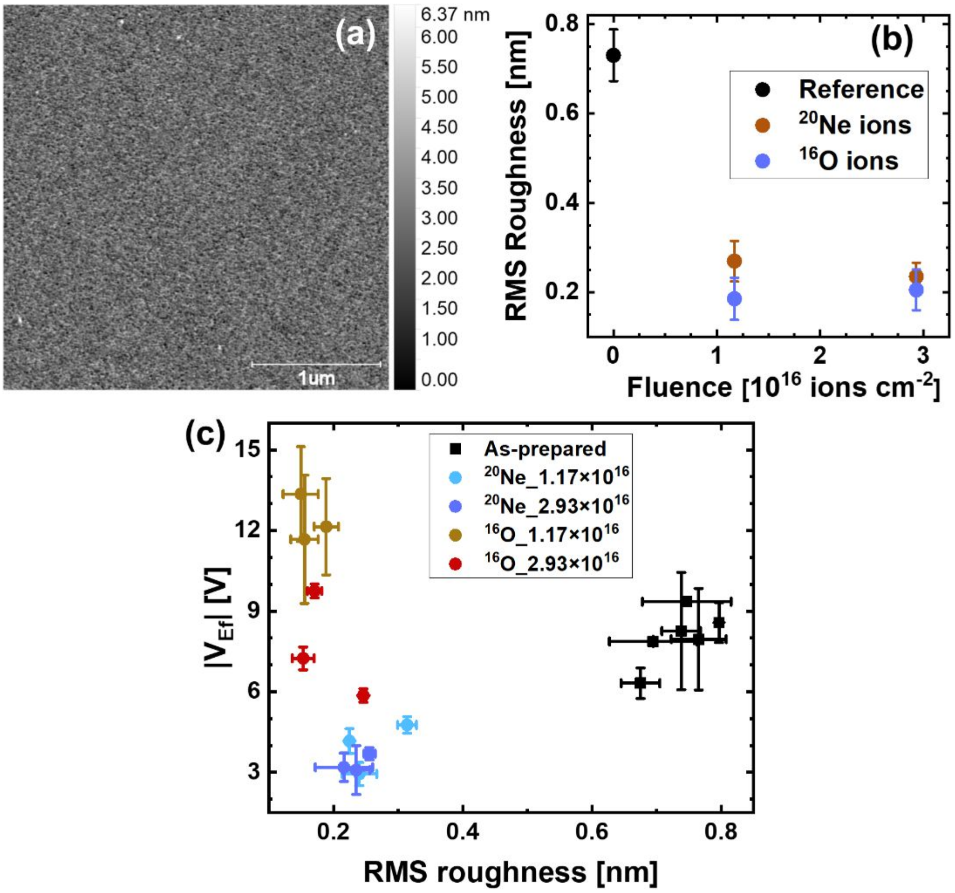

Figure S3. *(a) AFM image of the as-prepared TiO_2 layer; (b) RMS roughness of the TiO_2 surface as a function of fluence of 8 keV ^{20}Ne and ^{16}O ions implanted in the TiO_2 layer; (c) Scatter plot of RMS roughness of TiO_2 surface and electroforming voltage (V_{Ef}). Each point corresponds to 3-4 devices.*

Figure S3 shows (a) the Atomic Force Microscopy (AFM) image of the as-prepared TiO_2 surface, (b) root mean square (RMS) roughness of the TiO_2 surface as a function of the ion fluence, and (c) scatter plot of RMS roughness with electroforming voltage (V_{Ef}). The RMS roughness of the TiO_2 layer decreases after implantation with both ^{16}O and ^{20}Ne ions. Each point in Figure S3(c) corresponds to 3-4 devices with error bars showing the standard deviation in RMS roughness and V_{Ef}. Ion implantation reduces the roughness of the TiO_2 surface, and thus the interface roughness of the Pd(TE)-TiO_2. No systematic relation between Pd(TE)-TiO_2 interface roughness and V_{Ef} is observed in the present case.

Curve fits for Hard X-ray Photoelectron Spectroscopy (HAXPES) spectrum:

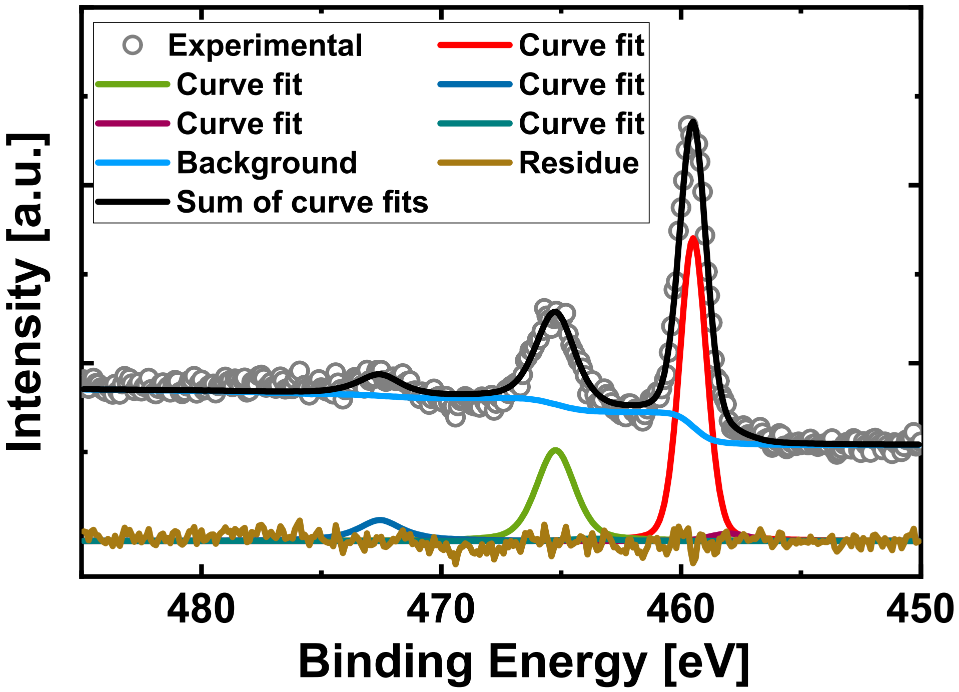

Figure S4. *HAXPES Ti 2p core level spectrum for the as-prepared sample, along with the curve fits for all the peaks, the sum of the fitted curves and the background.*

Electroforming in as-prepared and implanted samples:

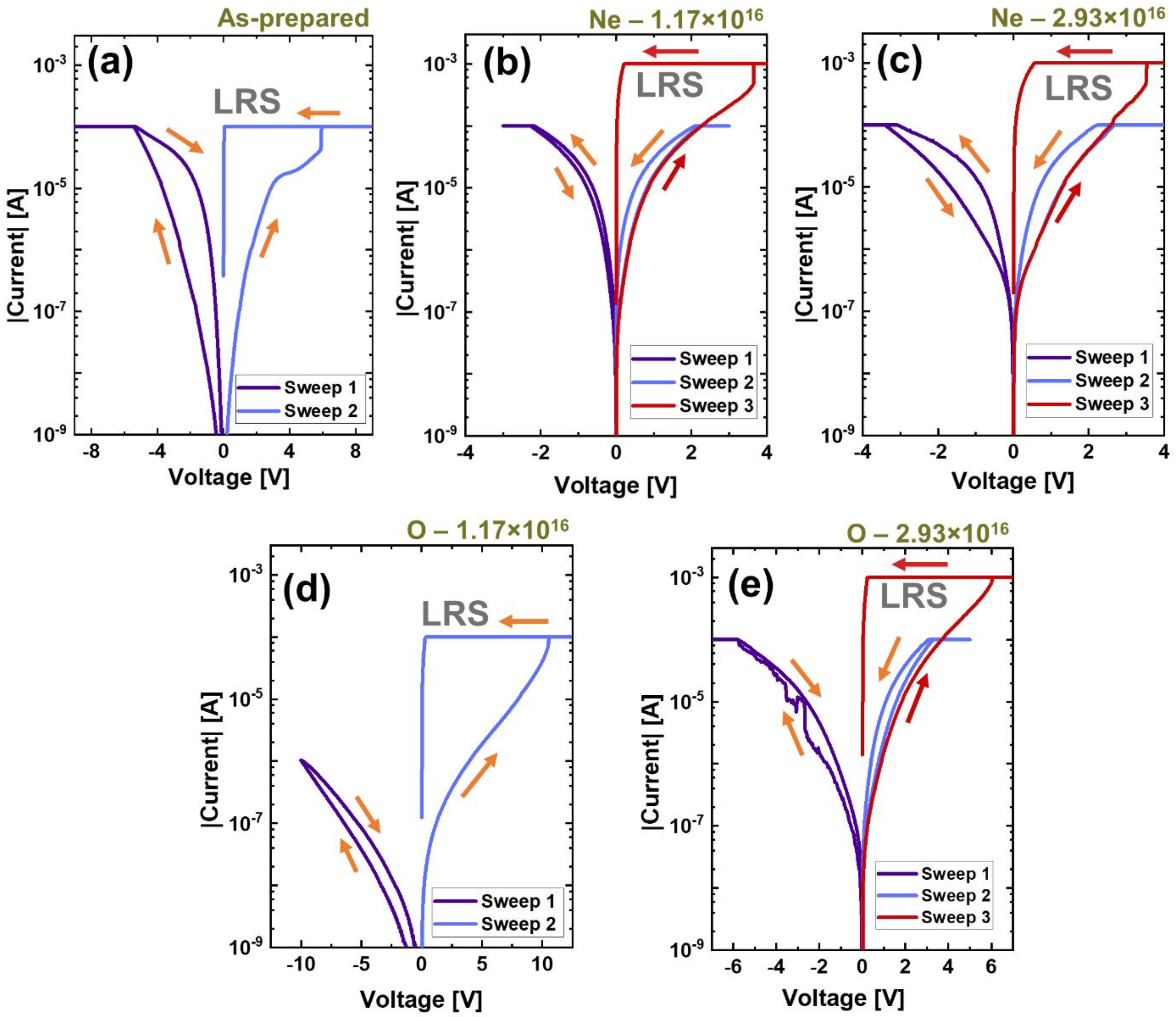

Figure S5. *Electroforming steps in (a) as-prepared sample, samples implanted with 8 keV ^{20}Ne at fluences of (b) 1.17 × 10^{16} and (c) 2.93 × 10^{16} ions cm^{-2} and samples implanted with 8 keV ^{16}O at fluences of (d) 1.17 × 10^{16} and (e) 2.93 × 10^{16} ions cm^{-2}.*

For the as-prepared sample, a voltage sweep from 0 V → -10 V → 0 V was applied to the top electrode (TE) at a compliance current (I_{cc}) of 0.1 mA, grounding the bottom electrode (BE). A decrease in the resistance of the as-prepared Pd/TiO_2/Pd samples is observed. Then, a voltage sweep from 0 to +10 V was applied to the TE at the same I_{cc} of 0.1 mA. A sudden decrease in the sample resistance (digital switching) is observed at (8 ± 2) V. After this, the sample is in a state of lower resistance, which is stable over time (measured by applying a constant voltage of 5 mV across the sample). The sample is referred to as 'electroformed', and the voltage at which digital switching is observed is called electroforming voltage (V_{Ef}). The electroforming of

$Pd/TiO_2/Pd$ samples requires at least two steps. **Figure S5**(a) shows a typical two-step electroforming process observed in as-prepared samples, where the first sweep is performed along the negative voltage. As the sample is symmetric, similar properties are expected if the first voltage sweep is across the positive voltage, as reported for $Pt/TiO_2/Pt$.[2]

The same procedure was followed for all categories of implanted samples: first sweep along the negative voltage, followed by a sweep along the positive voltage, both at an I_{cc} of 0.1 mA. Figure S5 shows the typical electroforming steps for samples implanted with 8 keV $^{20}Ne^{1+}$ ions at fluences of (b) 1.17×10^{16} and (c) 2.93×10^{16} ions cm^{-2} and 8 keV $^{16}O^{1+}$ ions at fluences of (d) 1.17×10^{16} and (e) 2.93×10^{16} ions cm^{-2}. Ne-implantation increases the concentration of oxygen vacancies ($V_O^{\cdot\cdot}$), reducing the initial resistance of the samples. As a result, the samples reach the I_{cc} of 0.1 mA before reaching V_{Ef}. The I_{cc} was increased to 1 mA to reach the V_{Ef} for the Ne-implanted samples. For O-implanted samples, the V_{Ef} can be reached at $I < 0.1$ mA at a fluence of 1.17×10^{16} ions cm^{-2}. However, for the sample implanted at a fluence of 2.93×10^{16} oxygen ions cm^{-2}, a current exceeding 0.1 mA was required to reach the V_{Ef} and the I_{cc} was subsequently raised to 1 mA to electroform the implanted samples.

IV characterisation after electroforming in as-prepared and implanted samples:

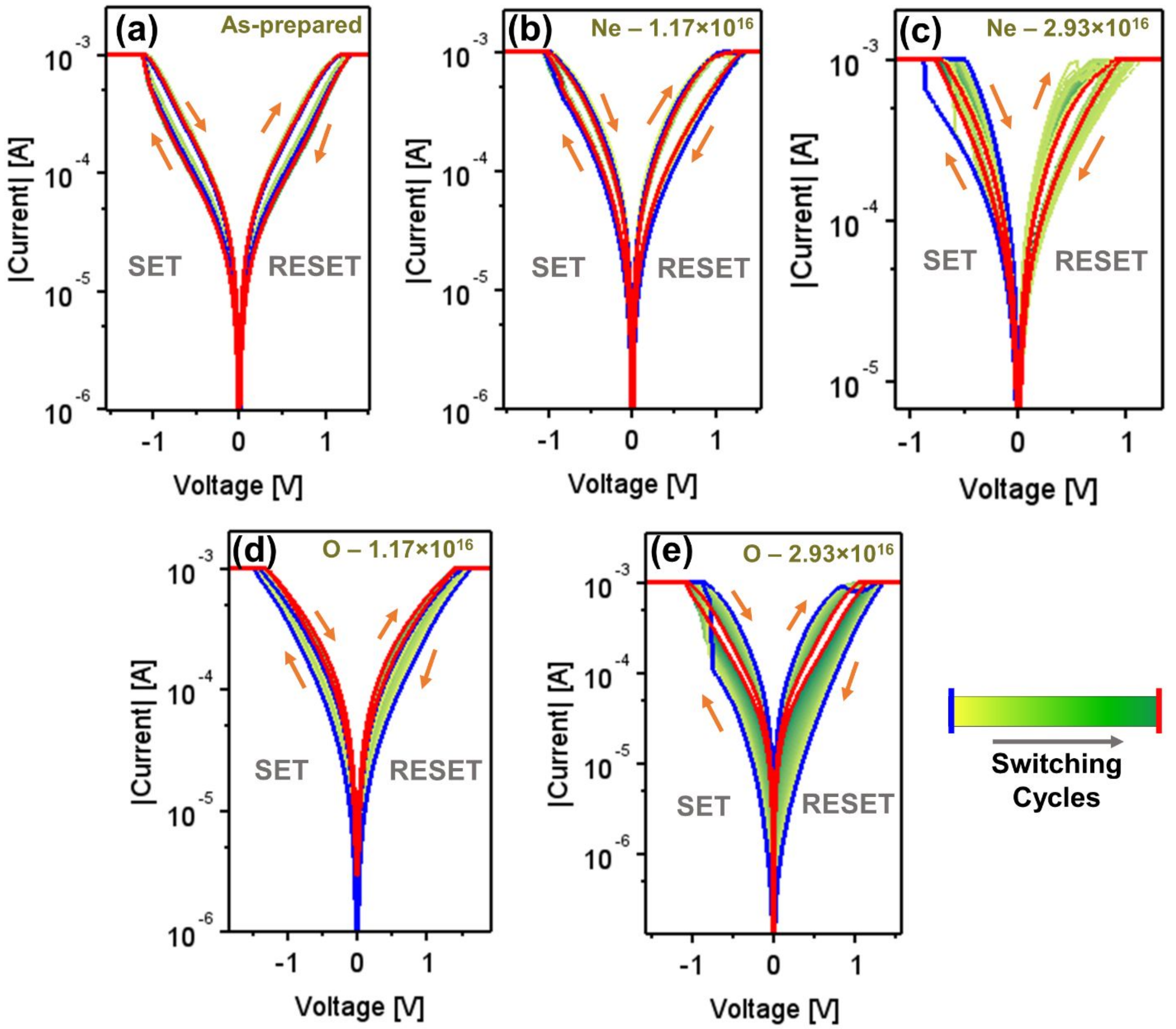

Figure S6. *IV characteristics, after electroforming, for (a) as-prepared sample, samples implanted with 8 keV ^{20}Ne at fluences of (b) 1.17 × 10^{16} and (c) 2.93 × 10^{16} ions cm^{-2} and samples implanted with 8 keV ^{16}O at fluences of (d) 1.17 × 10^{16} and (e) 2.93 × 10^{16} ions cm^{-2}.*

Figure S6 shows the typical IV curves after electroforming at I_{cc} = 1 mA for (a) as-prepared samples and samples implanted with 8 keV $^{20}Ne^{1+}$ ions at fluences of (b) 1.17×10^{16} and (c) 2.93×10^{16} ions cm^{-2} and 8 keV $^{16}O^{1+}$ ions at fluences of (d) 1.17×10^{16} and (e) 2.93×10^{16} ions cm^{-2}. All the samples exhibit analogous, bipolar resistive switching after electroforming. At an I_{cc} = 1 mA, all the samples can be 'SET' to a low resistance state (HRS) by applying a sweep along negative potential and 'RESET' to a high resistance state (LRS) by applying a sweep along positive potential to the top electrode (TE). After electroforming, the voltage polarity for the SET/RESET process is determined by the configuration of the CFs, rather than a change in

vacancy concentration profile in the TiO_2 layer.[2] **Figure S7** shows a scatter plot of the resistance ratio (R_{Ratio} = HRS/LRS) measured during the first few switching cycles (I_{cc} = 1 mA) for samples exhibiting RS after electroforming. On average, an increase in R_{Ratio} (at given I_{cc}) is observed with ion implantation.

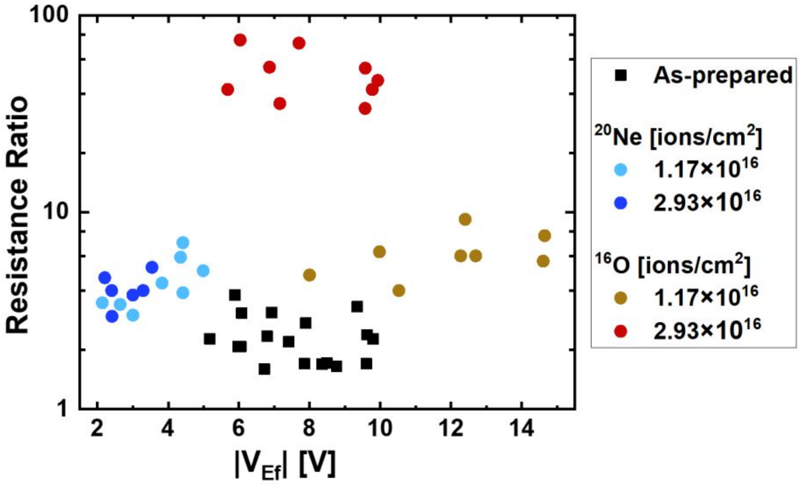

Figure S7. *Scatter plot showing resistance ratio (R_{Ratio} = HRS/LRS) measured during the first few switching cycles.*

References:

(1) Ziegler, J. F.; Ziegler, M. D.; Biersack, J. P. SRIM – The Stopping and Range of Ions in Matter (2010). *Nucl. Instrum. Methods Phys. Res. Sect. B Beam Interact. Mater. At.* **2010**, *268* (11–12), 1818–1823. https://doi.org/10.1016/j.nimb.2010.02.091.

(2) Jeong, D. S.; Schroeder, H.; Breuer, U.; Waser, R. Characteristic Electroforming Behavior in Pt/TiO2/Pt Resistive Switching Cells Depending on Atmosphere. *J. Appl. Phys.* **2008**, *104* (12), 123716. https://doi.org/10.1063/1.3043879.
